# Bone marrow-derived progenitor cells develop into myeloid-derived suppressor cells at metastatic sites

**DOI:** 10.1186/2051-1426-1-S1-P188

**Published:** 2013-11-07

**Authors:** Amber Giles, Yorleny Vicioso, Miki Kasai, Steven Highfill, Arnulfo Mendoza, Rosandra Kaplan

**Affiliations:** 1 Pediatric Oncology Branch, NCI, NIH, Bethesda, MD, USA

## 

The ability of tumor cells to metastasize to distant tissues is the most lethal aspect of cancer. Prior to detectable metastasis tumors elicit distant changes within the bone marrow and future sites of metastasis, including formation of the pre-metastatic niche. We have found that prior to detectable spontaneous metastasis, a primary tumor causes enhanced production and mobilization of progenitor cells from the bone marrow into the blood. We find in cancer patients that increased circulating progenitors are associated with metastatic disease progression. By utilizing bone marrow transplant models and a novel ex vivo lung culture, we demonstrate that bone marrow-derived progenitor cells accumulate at the primary tumor as well as metastatic lesions. Blood and bone marrow of tumor-bearing mice are able to form significantly more myeloid colonies in a CFU assay. As our cancer models approach detectable spontaneous metastases, we observe a loss of progenitor cells in pre-metastatic sites and a two- to four- fold increase in myeloid-derived suppressor cells (MDSCs). We further traced adoptively transferred bone marrow-derived progenitors in tumor-bearing mice and demonstrate that these cells contribute to the MDSC population. The MDSCs identified by immunophenotyping by flow cytometry are immunosuppressive and capable of suppressing T cell proliferation in response to anti-CD3/anti-CD28 bead stimulation. Together, these data suggest that myeloid-derived suppressor cells contribute to metastatic progression at distant sites. Further, bone marrow-derived progenitor cells can provide a prognostic marker for tumor progression and metastatic risk.

